# Two-Stage Total Knee Arthroplasty Revision With Extended Antibiotic Spacer Use

**DOI:** 10.7759/cureus.14854

**Published:** 2021-05-05

**Authors:** Clayton R Welsh, Patricia A Baumann

**Affiliations:** 1 Orthopaedics, University of Central Florida, Orlando, USA; 2 Orthopaedic Surgery, C.W. Bill Young Department of Veterans Affairs Medical Center, Saint Petersburg, USA

**Keywords:** joint infection, antibiotic spacer, two-stage revision, prosthetic joint, revision surgery, orthopedic surgery

## Abstract

Despite the many benefits of total knee arthroplasty (TKA) in the treatment of degenerative arthritis, infection of the total knee prosthesis presents a dangerous post-operative complication affecting 0.5-1.9% of all cases. Infection after the first three post-operative weeks is treated with either one or two-stage revision operations involving the removal of all prosthetic components. Two-stage revision operations are more commonly used and involve the removal of prosthetic components followed by the implantation of a cement mold infused with antibiotics (antibiotic spacer) as well as systemic antibiotic treatment for four to six weeks before prosthetic reimplantation. This case report details a TKA revision in a patient with osteoarthritis of the knee. The patient presented with an elevated erythrocyte sedimentation rate, C-reactive protein, and white blood cell count nearly two years after the primary operation and was found to have an infected total knee prosthetic. A two-stage revision was planned but due to scheduling disruption by the coronavirus disease 2019 pandemic, the second stage of the operation was delayed until 12 months after the stage one operation. The patient ambulated without pain on an antibiotic spacer for 12 months, providing information about the long-term use of spacers. This case also offers a look at a potential benefit to one-stage operations, which have been shown in the literature to have similar outcomes as two-stage operations. The patient had a medical history of psoriasis and immunosuppressive treatment with methotrexate, two risk factors for prosthetic joint infection, and may have benefited from prophylactic antibiotic therapy extending beyond the perioperative period. The goal of this case report is to detail the prolonged use of an antibiotic spacer, examine the risks and benefits of one and two-stage total knee revisions, and discuss prophylactic antibiotic use in high-risk patients following TKA.

## Introduction

Total knee arthroplasty (TKA) is frequently used for the definitive management of osteoarthritis and rheumatoid arthritis with over 600,000 cases performed in the United States each year and an expected growth to 1.26 million cases yearly by 2030 [1]. While there are many benefits to the procedure such as early mobility, decreased pain, and improved quality of life, several known post-operative complications can occur. Infection is a feared complication of joint replacement and occurs in 0.5-1.9% of primary TKAs and 8-10% of revision TKAs [2]. Risk factors for infection include age over 80, alcoholism, diabetes mellitus, renal or pulmonary insufficiency, systemic inflammatory disease, systemic immune compromise, and immunosuppressive drugs [3]. Treatment options for infected prosthetic joint include irrigation and debridement with retention of components, one-stage removal and replacement of prosthetic implants, or two-stage removal of the prostheses involving the insertion of an antibiotic spacer and later reimplantation of a new prosthetic device [2]. Infection within the first three weeks post-operatively can be treated with irrigation and removal of polyethylene components, while the removal of prosthetic components in a one or two-stage operation is needed to treat infection after the first three weeks [2].

The diagnosis of prosthetic joint infection is an evolving process that remains imperfect. An infected prosthetic knee most commonly presents with pain, and even without signs of inflammation such as erythema and warmth, a painful joint should be concerning for infection [2]. Key laboratory tests include erythrocyte sedimentation rate (ESR) and C-reactive protein (CRP), and a joint aspiration can aid in both the diagnosis of an infected joint and the identification of the specific pathogen. Joint aspirate should be evaluated for a white blood cell count above 17,000/uL, a neutrophil percentage greater than 65%, and the presence of identifiable pathogenic bacteria [4].

Two-stage operations are considered the ideal treatment for late prosthetic infection and involve removal of all prosthetic components including cement [2]. After aggressive irrigation and debridement, an antibiotic cement spacer is left in place and systemic antibiotics are administered for four to six weeks [2]. Reimplantation of the final prosthetic device takes place when the infection has cleared, the patient is medically fit, and the prior wound has healed [5]. Two-stage operations are considered ideal because they allow for more accurate identification of bacteria, guided antibiotic treatment, and increased confidence in infection eradication than one-stage procedures [2]. One-stage operations are frequently used in many European countries and have the benefit of faster treatment and recovery, decreased complications associated with antibiotic spacer use, and decreased morbidity associated with multiple operations [6]. A limitation of one-stage operations is that the identity and sensitivity of the infectious pathogen must be diagnosed pre-operatively [6].

The aim of this case report is to detail a two-stage revision operation following an infected total knee prosthetic. The report highlights several risk factors for prosthetic infection and shows how antibiotic spacers can provide adequate mobility and function beyond the typically recommended period of use, as 12 months passed between the first and second stages of this operation due to the coronavirus disease 2019 (COVID-19) pandemic.

## Case presentation

**Figure 1:**
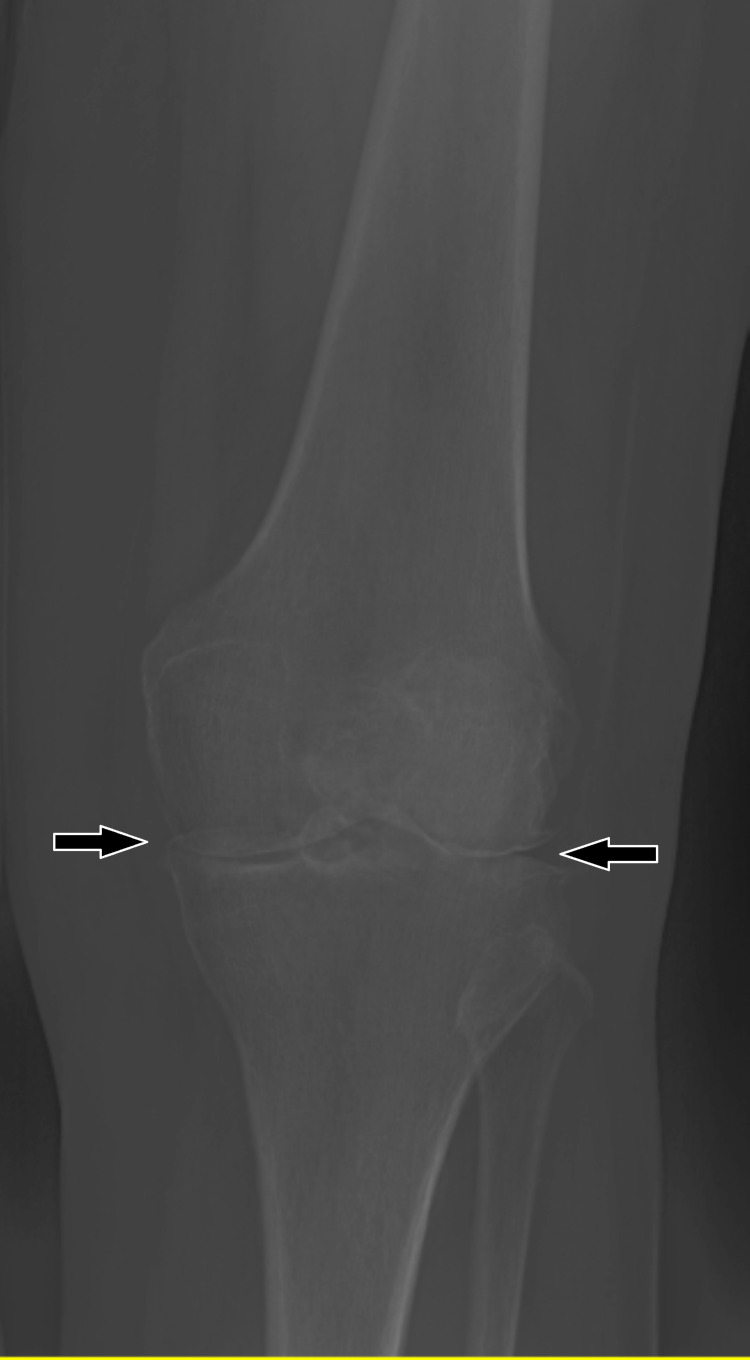
Left knee X-ray showing medial and lateral joint space narrowing consistent with osteoarthritis (black arrows).

The patient described in this report first presented to the orthopedic clinic in 2014 at the age of 63. She complained of a dull, constant, and non-radiating pain in her left knee that intensified with activity and limited ambulation. Magnetic resonance imaging was performed and showed tri-compartment degeneration, and the patient was diagnosed with osteoarthritis of the left knee. The patient’s relevant past medical history included psoriasis managed with methotrexate, calcipotriene, and halobetasol propionate cream. Prior knee surgery included a right total knee arthroscopy performed in 2013 due to osteoarthritis. She was treated conservatively with two Hylan G-F 20 injections one year apart, which she tolerated well with reported decreased pain. After 18 months, the patient stated the injections had lost efficacy and she would like to consider a TKA for definitive treatment. Imaging performed at this time showed significant osteoarthritis (Figure 1).

The patient was scheduled for left TKA three years after her initial left knee pain complaint. Methotrexate was held one week prior to surgery. Her left TKA followed an uncomplicated operative course under general anesthesia. A sterile tourniquet was inflated to 275 mmHg, the knee was prepared and draped, and 2 g of cefazolin was administered. A medial parapatellar approach was taken with electrocautery used to achieve hemostasis. The hypertrophic synovium was debrided. The menisci, posterior cruciate ligament, and anterior cruciate ligament were debrided and the distal femoral cut made followed by the tibial cut. The implants were sized with good stability in flexion and extension reported. An Attune Primary Total Knee System was installed (DePuy Synthes Companies, Massachusetts). High-viscosity methylmethacrylate cement was used. The quadriceps, subcutaneous tissue, and skin were closed and a sterile dressing was applied. The immediate post-operative course was uncomplicated, and the patient was discharged on post-operative day six. Her two-week, four-week, three-month, five-month, and one-year visits were without complications and the patient reported no pain, adequate range of motion, and walking, dancing, and exercising as desired.

Two years after the left TKA operation, the patient presented with ongoing discomfort in her left knee. Laboratory tests at this visit revealed a white blood cell count of 7,700/uL, CRP 14.6 mg/L, and ESR 89 mm/hour. A joint aspiration performed at this time revealed 14,482 nucleated cells with 78% neutrophils. Her examination showed mild medial/lateral laxity with a large effusion around the left knee. There was no anterior/posterior instability, but imaging showed loosening of the tibial baseplate. A methicillin-resistant *Staphylococcus aureus* nasal swab was negative. A diagnosis of infected left total knee prostheses was made, and the patient was scheduled for left total knee antibiotic spacer insertion with a Biomet Spacer One and staged revision TKA (Zimmer Biomet, Indiana).

**Figure 2:**
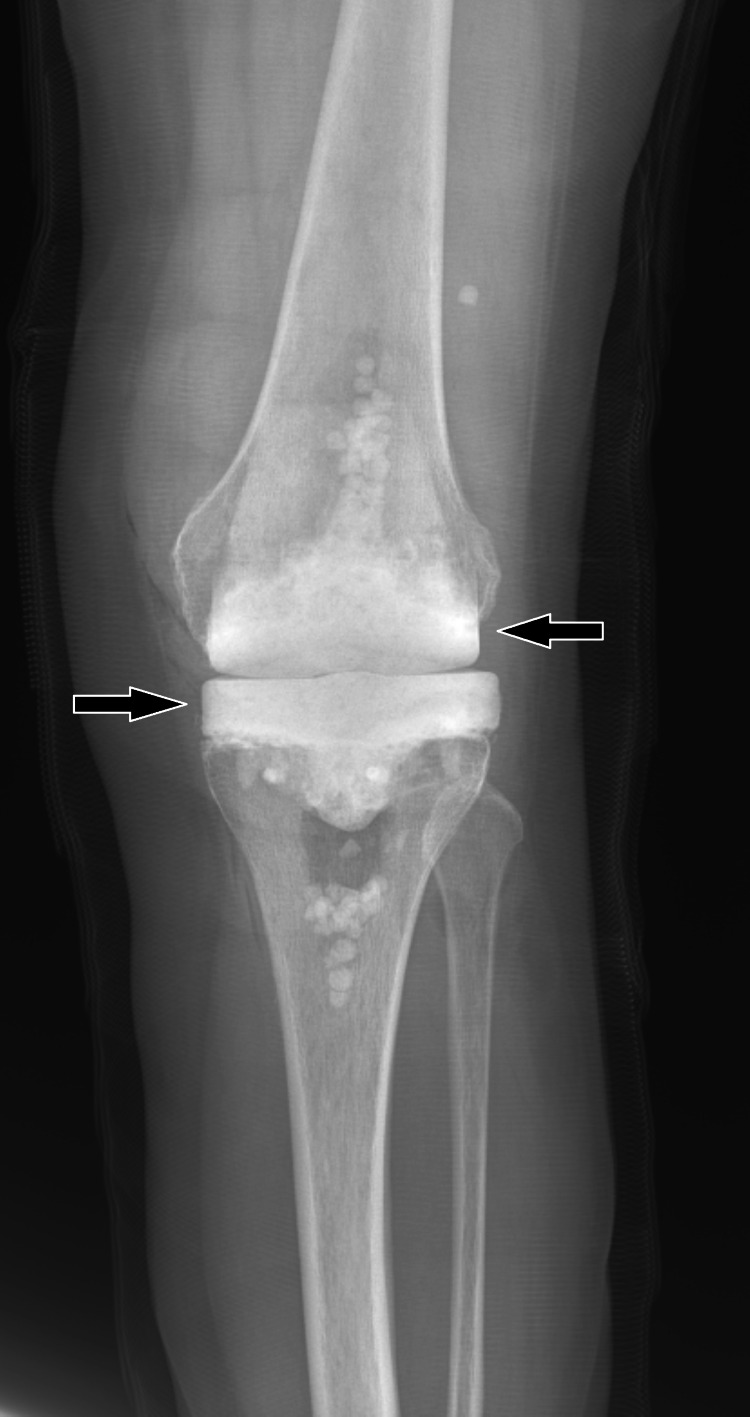
Left knee X-ray showing femoral and tibial spacers created from Cobalt Bone Cement with Gentamicin (black arrows).

Stage one left total knee arthroscopic revision commenced in the fall of 2019. Methotrexate was held one week prior to surgery. The left knee was prepped and draped in the usual sterile fashion and general anesthesia was used with cefazolin and vancomycin administered. A sterile tourniquet was used and hemostasis was achieved through electrocautery. A medial parapatellar approach following the previous incision was used. The knee was flexed to 90 degrees and the poly insert was removed. The femoral component was removed using the small oscillating saw, punch, and mallet. The tibial component was removed next using the small oscillating saw, flexible osteotome, and extraction device. All remaining cement was removed. Sizing of the tibia and femur was performed, and cement molds were created. The cement mold was created from Cobalt Bone Cement with Gentamicin. One gram of vancomycin per cement bag was added. Two bags of cement were used to create the femoral component, two bags to create the tibial component, and one bag for fixation leading to a total of five cement bags with 5 g of vancomycin added. The knee was irrigated with Dakin’s solution, chlorhexidine, peroxide, and betadine as well as 6 L of antibiotic pulse lavage followed by 3 L of plain pulse lavage. Antibiotic beads were placed in the canals and posterior knee. The cement spacer was inserted and cemented into place. The knee was again irrigated, and the quadriceps, subcutaneous tissue, and skin were closed. The wound was dressed in a sterile fashion, and immediate post-operative X-rays confirmed the cement inserts were in place, as can be seen in Figure 2.

The patient’s immediate post-operative course was uncomplicated with no signs of continued infection. On post-operative day three, a peripherally inserted central catheter was inserted, and cefazolin antibiotic treatment continued for two months. The patient was discharged on post-operative day six with adequate pain control and after meeting physical therapy goals. The patient reported no complications at two-week, five-week, two-month, and three-month visits. The patient was scheduled to undergo her second-stage operation in the spring of 2020. However, due to the COVID-19 pandemic, non-traumatic orthopedic surgeries were placed on hold and the patient was informed that her surgery would be delayed. In the fall of 2020, when restrictions on orthopedic procedures were lifted, her surgery was scheduled. The patient was seen in the office two months prior to her operation and reported no difficulty ambulating, though moderate pain and discomfort persisted, not limiting her daily activities. On examination, she had a well-healed incision, adequate patellar tracking, 20 degrees of extension, 110 degrees of flexion, and 4/5 strength in flexion and extension with no sign of infection, erythema, or effusion. Her knee was aspirated at this time and showed no growth or signs of infection. Imaging showed a left knee cement spacer in place with no evidence of loosening or shifting.

**Figure 3:**
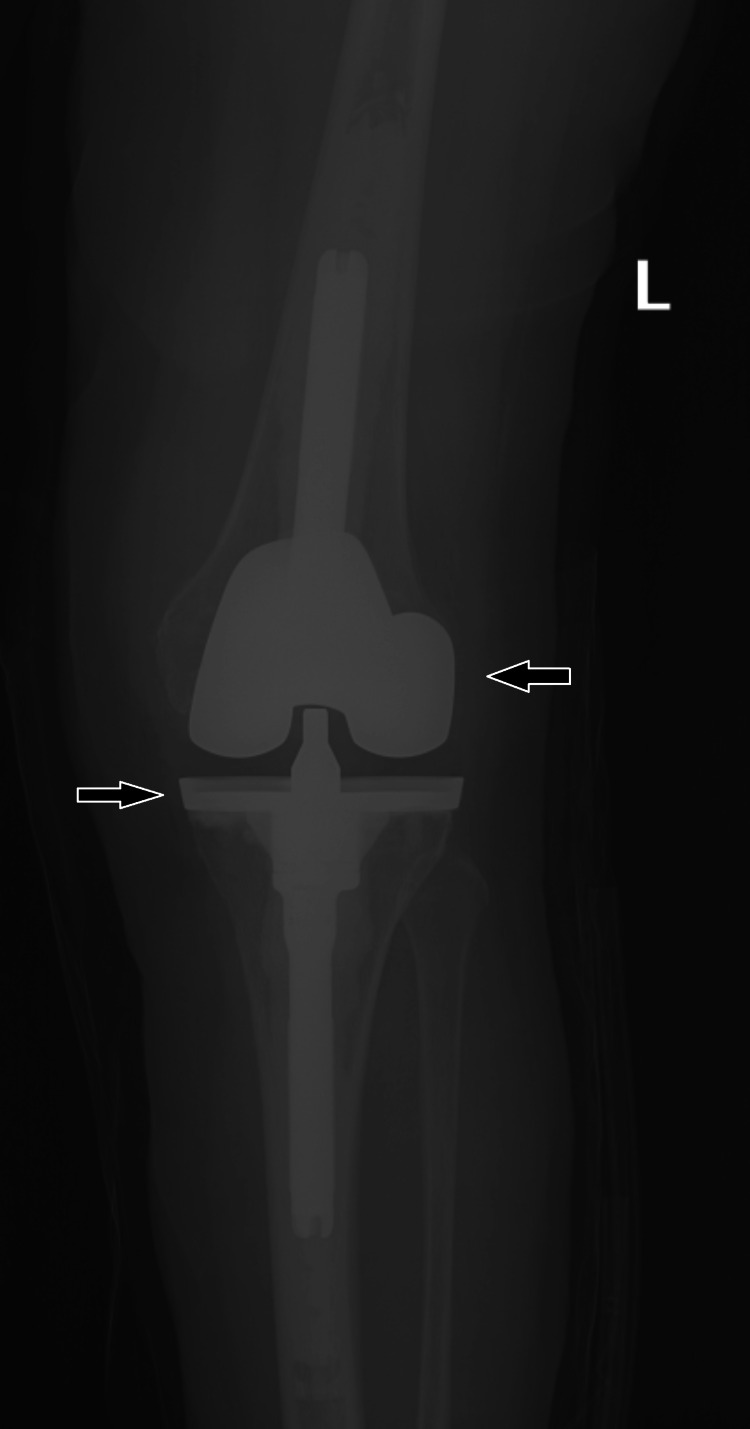
Left knee X-ray showing a DJO Surgical Total Knee Arthroplasty System (black arrows).

The second stage of the patient’s left TKA revision was completed in the fall of 2020. Her pre-operative laboratory work showed a white blood cell count of 5,700/uL, ESR of 32 mm/hour, and CRP of 3.6 mg/L. General anesthesia was used, and the left knee was draped in the usual orthopedic fashion. A sterile tourniquet was inflated, and hemostasis was achieved through electrocautery. A median parapatellar approach through the prior incision was taken. The knee was flexed to 90 degrees and the cement spacer was removed. Frozen sections were extracted and sent to the pathology department for evaluation. Following confirmation from the frozen section of less than five white blood cells per high-powered field, vancomycin and cefazolin were administered and the reimplantation commenced. The femur and tibia were appropriately sized and the bone cuts were freshened. The knee was irrigated with antibiotic pulse lavage, chlorhexidine, peroxide, Dakin’s solution, and betadine. A pathology report on the permanent section was reviewed during the operation and showed less than five white blood cells per high-powered field. Prosthetic reimplantation commenced with a DJO Surgical Total Knee Arthroplasty System (DJO Surgical, Texas). Adequate range of motion and proper patellar tracking was achieved. The knee was irrigated, and the quadriceps tendon, subcutaneous tissue, and skin were closed. The knee was dressed with a sterile dressing. X-rays showed the implant to be in an adequate position (Figure 3). The patient had an uncomplicated immediate post-operative course and was discharged on post-operative day three with enoxaparin and oxycodone prescriptions after adequate pain control and physical therapy goals were achieved. The patient was prescribed 100 mg doxycycline to be taken daily for life for the prevention of joint infection. One and two-week post-operative visits revealed no abnormalities with a gradual return to function.

## Discussion

The patient presented in this case report provides an interesting examination of the prolonged use of an antibiotic cement spacer for reasons other than a continued infection. The timing between stage one and stage two of a revision TKA is typically two to three months, allowing the infection to be treated by antibiotics and the previous surgical wound to heal. Due to the COVID-19 pandemic, the stage-two operation for this patient was not undertaken for one year after the initial placement of the antibiotic spacer. Follow-up visits with the patient confirmed that the patient did not suffer from any acute pain, instability, or reinfection during the one-year period. The durability of antibiotic cement spacers has been studied in a limited nature; however, data have shown successful retention of antibiotic spacers for up to six years [7]. The patient presented in this report offers insight into the nature of extended use of antibiotic cement spacers beyond the recommended replacement period, and the lack of complications seen in this case provides evidence that spacers may occasionally be retained safely under exceptional circumstances.

Despite the consequences of prosthetic joint injection, there is no consensus on the use of prolonged post-operative prophylactic antibiotic medication. After revision of a TKA due to infection, patients are often placed on a lifelong suppressive antibiotic medication, which studies have shown to be effective in up to 84% of patients [8]. In the case described, the patient was discharged from the hospital with a prescription for lifelong doxycycline 100 mg daily. The patient presented to the orthopedic team with a known diagnosis of psoriasis treated with methotrexate. As the underlying systemic disease and immunosuppressive medication are two significant risk factors for prosthetic infection, there could have been a potential benefit to starting the patient on lifelong doxycycline following her primary revision. Recent data have supported the use of prophylactic antibiotics in high-risk patients, with those not given prophylactic antibiotics five times as likely to develop infection [9]. Prophylactic antibiotics may have reduced the risk of prosthetic joint infection in this high-risk patient, though the risks of unneeded antibiotic treatment are not negligible. Further research is currently needed to address the role of extended post-operative prophylactic antibiotics in high-risk TKA patients.

While two-stage revision operations are considered the gold standard for the treatment of prosthetic infections, single-stage operations are increasing in frequency as results show favorable outcomes when examining mortality, cost, and length of hospitalization [10]. Even without the period of prolonged intra-articular antibiotics used in two-stage revisions, the rate of reinfection for one-stage operations in recent literature has been reported as 7.6% compared to 8.8% for two-stage operations while the average infection eradication rate was 87.1% for the one-stage procedure and 84.8% for the two-stage procedure with similar functional outcomes across both types of the procedure [11,12]. In the case of the patient presented, it would appear that a one-stage operation may have been beneficial, provided pre-operative cultures revealed the identity and sensitivity of the pathogen. While the patient’s cement antibiotic spacer did not cause excessive pain or instability, given its prolonged retention period, the patient likely would have preferred early definitive management. Additionally, the requirement of tapering methotrexate prior to multiple surgeries could have worsened the patient’s underlying psoriasis and have been avoided with a one-stage procedure. Though the COVID-19 pandemic was an unforeseeable event at the time of the initial revision operation, one-stage revisions could be considered for the effective treatment of infected total knee prosthetics in cases with questionable patient follow-up or where multiple operations could cause a significant medical risk to the patient.

## Conclusions

The case described in this report details a prolonged two-stage revision process for an infected total knee prosthetic. It highlights the function of an articulating antibiotic spacer beyond the typically recommended period and shows how a spacer can allow for effective ambulation for up to 12 months. This case demonstrates the potential benefits of one-stage revision operations, as the second stage was significantly delayed due to the COVID-19 pandemic, and it outlines a reason why one-stage operations may be preferred for patients who may be lost to follow-up prior to the second operation. A one-stage operation may also be beneficial for a patient with medical contraindications to multiple surgeries. Finally, the need for definitive prophylactic post-operative antibiotic treatment guidelines is paramount, especially in patients with multiple risk factors for joint infection.
